# Decoding reach-to-grasp from EEG using classifiers trained with data from the contralateral limb

**DOI:** 10.3389/fnhum.2023.1302647

**Published:** 2023-11-08

**Authors:** Kevin Hooks, Refaat El-Said, Qiushi Fu

**Affiliations:** ^1^Mechanical and Aerospace Engineering, University of Central Florida, Orlando, FL, United States; ^2^College of Medicine, University of Central Florida, Orlando, FL, United States; ^3^Biionix Cluster, University of Central Florida, Orlando, FL, United States

**Keywords:** electroencephalography, brain-machine interface, decoding, reaching, grasping, visuomotor transformation

## Abstract

Fundamental to human movement is the ability to interact with objects in our environment. How one reaches an object depends on the object’s shape and intended interaction afforded by the object, e.g., grasp and transport. Extensive research has revealed that the motor intention of reach-to-grasp can be decoded from cortical activities using EEG signals. The goal of the present study is to determine the extent to which information encoded in the EEG signals is shared between two limbs to enable cross-hand decoding. We performed an experiment in which human subjects (*n* = 10) were tasked to interact with a novel object with multiple affordances using either right or left hands. The object had two vertical handles attached to a horizontal base. A visual cue instructs what action (lift or touch) and whether the left or right handle should be used for each trial. EEG was recorded and processed from bilateral frontal-central-parietal regions (30 channels). We trained LDA classifiers using data from trials performed by one limb and tested the classification accuracy using data from trials performed by the contralateral limb. We found that the type of hand-object interaction can be decoded with approximately 59 and 69% peak accuracy in the planning and execution stages, respectively. Interestingly, the decoding accuracy of the reaching directions was dependent on how EEG channels in the testing dataset were spatially mirrored, and whether directions were labeled in the extrinsic (object-centered) or intrinsic (body-centered) coordinates.

## 1. Introduction

In activities of daily living, an important motor function is reaching and interacting with objects of interest. This ability can be severely limited in patients with upper-limb motor impairment, such as stroke survivors and individuals with spinal cord injuries. Advances in brain-machine (BMI) or brain-computer interfaces have provided the technological foundation to decode information from neural signals associated with cortical activities to drive assistive robots or support rehabilitation (Lebedev and Nicolelis, 2017; López-Larraz et al., 2018). One of the extensively studied neural signal sources is electroencephalography (EEG) due to its high temporal resolution, non-invasiveness, and portability (Al-Quraishi et al., 2018; Orban et al., 2022). EEG-based BMI can be generally categorized into two types or a mix of these two types: *exogenous* and *endogenous* (Abiri et al., 2019). The exogenous EEG BMI is driven by cortical activities evoked by external stimuli, such as P300 signals (Fazel-Rezai et al., 2012) and steady-state visual-evoked potentials (Zhang et al., 2021). In this framework, the BMI can detect a user’s overt visual attention on a screen by matching EEG signals to particular waveform shapes or frequencies that are direct results of the stimuli. Therefore, it has great potential in creating communication interfaces such as BMI spellers (Rezeika et al., 2018). In contrast, the endogenous EEG BMI is driven by neural activities that are modulated by spontaneous motor intent as the users attempt to execute or imagine a specific motor action (Pereira et al., 2017). This type of BMI is believed to be more intuitive to use in movement control applications because the motor intent can be directly mapped to a similar action of the assistive or rehabilitation devices.

The endogenous EEG BMI is built with the assumption that the spatial, temporal, and/or spectral characteristics of EEG signals are associated with certain aspects of the intended movement. For reaching and object interaction, previous research has demonstrated that information about upper limb movement direction and type can be decoded from EEG with various levels of accuracy (Hammon et al., 2008; Wang and Makeig, 2009; Iturrate et al., 2018; Ofner et al., 2018; Xu et al., 2021, see Tables 1, 2). However, most of the existing studies have focused on the decoding of movement intent within the dominant limb (mostly in right-handed individuals) with only a few exceptions. Lew et al. (2014) investigated reaching direction decoding for left and right limbs in a couple of participants. They found no difference between the decoding accuracies associated with two limbs, but the channels that contributed most to the target discrimination were different between left-hand and right-hand decoders. A recent study found no difference between the accuracies of decoding grasp types between two hands, although the distribution of cortical activity patterns revealed significant lateralization for each hand (Schwarz et al., 2020). These results are consistent with the rich evidence that demonstrates the lateralization of motor control circuits for each limb (Serrien et al., 2006; Walsh et al., 2008; Sainburg, 2014; Schmitz et al., 2019). However, it is largely unclear the extent to which information encoded in EEG signals is limb-dependent and the extent to which two limbs can share the same BMI decoder for detecting movement intent.

**TABLE 1:** Summary of previous within-hand results of decoding hand actions.

References	Task	N_CH_	Classifier design	Window size	Task phase	Accuracy
Iturrate et al., 2018	Self-paced and selected power or precision grasp	15	1–6 Hz, 2-class LDA	0.5 s	Planning	69%
				1 s	Planning + Execution	78%
Ofner et al., 2018	Delayed onset with task cue, four grasp types	59	0.3–3 Hz, 2-class LDA	1 s	Execution	72%
Schwarz et al., 2020	Delayed onset with task cue, two grasp types	58	0.3–3 Hz, 2-class LDA	1 s	Execution	68%
Guo et al., 2019	Delayed onset with task cue, two grasp orientations	64	0.1–40 Hz, 2-class SVM	0.01 s	Planning	60%
				0.01 s	Execution	65%
Jochumsen et al., 2016	Cued force production with three grasp types	25	0.01–5 Hz, 2-class LDA	1 s	Planning	57%
Yong and Menon, 2015	Cued movement type, grasp vs. elbow motion	25	0.01–5 Hz, 2-class LDA	2 s	Motor imagery	61%

N_CH_ denotes the number of EEG channels used in the classifiers. Note that accuracy numbers are rounded, and only the means are listed.

**TABLE 2:** Summary of previous within-hand results of decoding reaching directions.

References	Number of directions	N_CH_	Classifier design	Window size	Task phase	Accuracy
Hammon et al., 2008	3	256	Broad band, logistic classifier	0.3 s	Planning	60%
				0.5 s	Execution	70%
	4	64	Broad band, logistic classifier	0.5 s	Planning	58%
Wang and Makeig, 2009	3	128	0–25 Hz, SVM	0.3 s	Planning	80%
Lew et al., 2014	4	64	0.1–1 Hz, LDA	0.25 s	Planning + Execution	75%
Wang et al., 2021	2	24	0.01–4 Hz, SVM	1 s	Execution	80%
Kim et al., 2019	2	64	0–40 Hz, SVM	0.3 s	Planning	79%
Sagila and Vinod, 2022	2	14	Broad band, SVM	2.5 s	Motor imagery	73%

N_CH_ denotes the number of EEG channels used in the classifiers. Note that accuracy numbers are rounded, and only the means are listed.

Reach-to-grasp movements involve complex visuomotor transformations from visual space to motor space (Davare et al., 2011). Task information is initially encoded in extrinsic object-centered spatial coordinates, whereas the motor output is encoded in intrinsic, effector-centered coordinates (Filimon, 2010). For instance, to retrieve a cup of coffee from the same location on the right side of the body, the left and right arms must produce different reaching kinematics and muscle activations but both hands may perform the same grasp action (power grasp). In this example, the encoding of grasping action can be shared across two limbs in both extrinsic and intrinsic coordinates, but the encoding of reaching may only be shared across two limbs in extrinsic coordinates. Non-human primate studies using cortical implants have revealed that many neurons in the frontoparietal network could encode movement information that is independent of the limb performing the movement, whereas some neurons are more tuned to limb-specific information (Kakei et al., 1999, 2001; Cisek et al., 2003; Chang et al., 2008). These studies imply that cortical activities could be associated with both extrinsic and intrinsic information in humans, but how neuronal activities manifest as EEG signals remains to be investigated.

The present study examines EEG-based decoding of reach-to-grasp behavior by asking human participants to interact with an object with either one of their hands. The object affords different interactions that could be similar in extrinsic or intrinsic coordinates. We tested the hypothesis that both the reaching direction and hand action type are encoded in EEG signals in a limb-independent fashion, thus allowing the same linear classifiers to operate above chance level for both hands.

## 2. Materials and methods

### 2.1. Participants

Ten young adult participants (4 M, 6 F. Mean age 23.4) enrolled in the study were all self-reported right-hand dominant. They had normal or corrected-to-normal vision, and no history of musculoskeletal or neurological disorders. All subjects were naïve to the purpose of the study and gave informed consent to participate in the experiment. The experimental protocols were approved by the Institutional Review Board at the University of Central Florida in accordance with the Declaration of Helsinki.

### 2.2. Experimental setup

**FIGURE 1:**
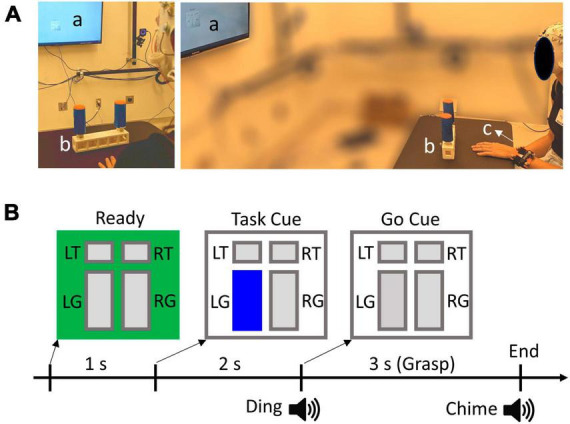
**(A)** Experimental set up. a, b, and c denote visual cue display, target object, and wrist tracker, respectively. **(B)** Experimental trial design and visual cue example. L and R denote left and right handles, whereas T and G denote Touch and Grasp conditions, respectively. In this example, the participant should grasp and lift the left handle, as indicated by the highlighted “LG” rectangle.

The participants sat comfortably in front of a table where a U-shaped object is located approximately 0.5 m from the participant’s body. The object weighs approximately 900 g and it has two vertical cylindrical handles attached to a rectangular base (Figure 1A). The distance between the two handles is 19 cm. The handles can be interacted with in two ways: touching the top with the index finger, or grasping with all fingers and lifting the object while keeping the object balanced. Therefore, there were 4 possible conditions to be performed by each hand (2 Actions × 2 Directions): Grasp Left handle, Touch Left handle, Grasp Right handle, Touch Right handle. The exact condition for each trial was given by visual cues using a customized LabView (National Instruments, Austin, TX) program, displayed on a monitor located 2 m in front of the participants. The visual cue has a size of 10 cm × 10 cm and consists of four rectangles indicating different conditions (Figure 1B). The participants were instructed to always focus their gaze on the visual cue, which is at about eye level, during the entire duration of each trial.

A 64-channel EEG ActiCap system (Brain Products, Germany) was individually fitted to each participant’s head size to ensure a proper connection of the electrodes. Conductive gel was applied to each electrode to achieve an impedance level below 20 kOhms. The electrode placement follows the international standard extended 10–20 system (Nuwer et al., 1998). Additionally, horizontal and vertical EOG channels were added to help remove ocular artifacts from EEG. The horizontal EOG electrodes were placed laterally next to each eye, and the vertical channels were placed above and below the right eye. EEG and EOG signals were recorded with BrainAmp at a sampling rate of 500 Hz. The movement of the wrists and the object were recorded at 120 Hz using an optical motion tracking system (OptiTrack, Corvallis, OR, USA). Reflective markers were attached to the object and both wrists of the participants (Figure 1A). The EEG and movement recordings were synchronized by event triggers generated by the LabView program.

### 2.3. Experimental procedure

The participants start each trial with one of their hands placed flat on the table in front of the center of the object. The color of the visual cue square turns green at the beginning of each trial instructing the participants to get ready. After 1 s, one of the rectangles corresponding to the target task condition was highlighted as a “Task” cue (Figure 1B). Subsequently, a “Go” cue was given 2 s after the Task cue, which is defined as the highlighted rectangle changing to gray coupled with a “ding” sound. Participants were asked to perform the desired action after the Go Cue with a natural speed. For Grasp conditions, the participants should grasp the target handle, lift the object a few inches off the table, and keep the object balanced. They must replace the object back to the table following a chime sound (3 s after Go). Note that the left and right handles of the object require opposite hand and wrist actions to balance the object because of the geometry and weight distribution. For example, the right handle requires a compensatory torque in the clockwise direction, which corresponds to the supination and pronation for the right and left hands, respectively. For Touch conditions, the participants placed the pad of the index finger on top of the target handle and moved their hands back after the chime sound (2 s after Go). The longer duration of the Grasp trials than the Touch trials was to ensure participants had sufficient time to complete the required action. After 20 trials of familiarization with the task conditions, participants performed a total of 8 trial blocks. Each block consisted of 40 randomized trials with each of the four conditions presented 10 times. Participants were instructed to switch hands between blocks, and each block was performed by the same hand. There was a total of 40 left-hand trials and 40 right-hand trials for each condition.

### 2.4. Data processing

The reflective markers were grouped into marker sets and formed rigid bodies whose centers were used to estimate the movement of the wrists in MATLAB (Natick, MA, USA). The 3D trajectories of the rigid-body centers were interpolated to account for missing points (<2% of total samples), followed by zero-lag smoothing with a 4-th order 5 Hz low-pass filter. Subsequently, the trajectory data was differentiated to calculate wrist movement velocities, which was then downsampled to 50 Hz. We define movement onset as the time when the wrist velocity is first above a threshold of 0.01 m/s and remains above the threshold for more than 0.5 s.

**FIGURE 2:**
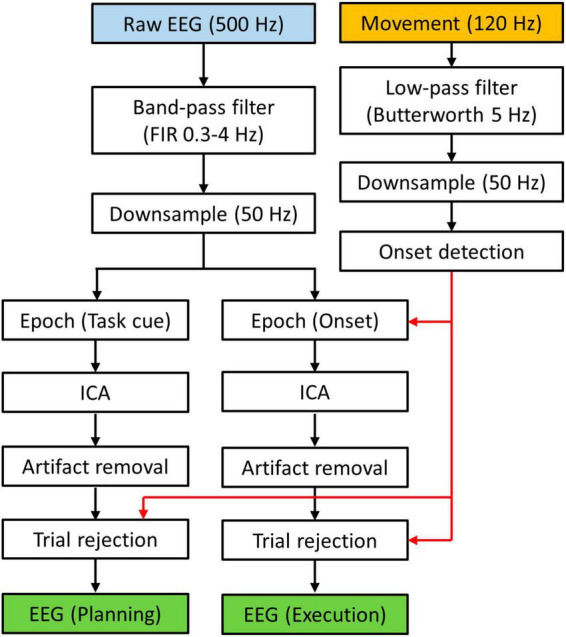
Data processing flow chart. EEG data was segmented to form two data sets. Movement data was used to determine which trial should be rejected.

All EEG data processing was performed in MATLAB using EEGLAB features (Delorme and Makeig, 2004). The EEG data was first re-referenced (Common Average) and bandpass filtered from 0.3 to 4 Hz with a zero-phase Hamming-windowed sinc FIR filter (Widmann et al., 2015). This low-frequency range has been shown to contain information about reach-to-grasp behaviors in previous studies (see Tables 1, 2). The filtered data was then downsampled to 50 Hz. We segmented the data in two ways (Figure 2). The first dataset was defined as epochs from the Ready cue to the Go cue, i.e., [−1, 2] s with respect to the Task cue. This dataset was used for investigating the planning phase (no movement) of the trials. The second dataset was defined as epochs of [−1, 2] s with respect to the movement onsets that were defined in the wrist movement analysis. This dataset was used for investigating the execution phase of the tasks. For both datasets, we implemented independent component analysis using AMICA (Palmer et al., 2012) and subsequently performed artifact removal using ADJUST algorithm (Mognon et al., 2011) with EOG signals as correlates. As the final step of the EEG processing, we rejected epochs based on movement data. Specifically, a trial is excluded from the planning dataset if the movement onset occurred before 0.5 s prior to the Go cue, leading to a trial rejection rate of 1.1 ± 1.0%. The rejected trials were considered to have movement occurred too early, which would generate execution-related cortical activities that should not occur in the planning phase. For the execution dataset, we rejected all trials that were excluded from the planning dataset with additional rejections if a trial had a movement onset before “Go” cue, or if participants did not move during a trial. This led to a trial rejection rate of 3.0 ± 1.6%. These rejected trials were considered to have movement occurred too early (which may cause substantial corrective movements during task execution), or a lack of execution stage. Both scenarios can make the neural activity of the trial an outlier for the execution dataset.

### 2.5. Classifier design

**FIGURE 3:**
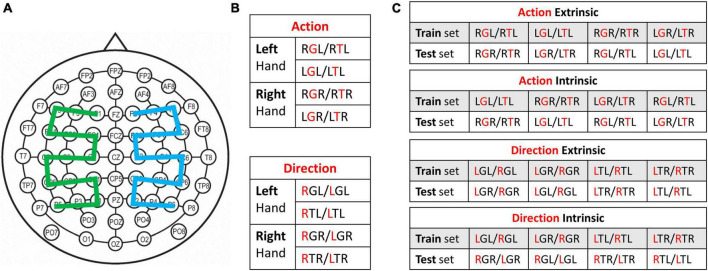
**(A)** Standard electrode montage used for classification. For mirrored montage, the green and blue regions are flipped with respect to the midline. **(B)** Within-hand classification setup. Cross-validation was used for each pair of conditions. **(C)** Cross-hand classification setup. Two conditions from one hand were used for training and two conditions from the other hand were used for testing. For Panels **(B,C)**, the red letter in the three-letter condition code represents the factor being classified. Each table represents a set of four classifiers that decode the same information, i.e., hand action or reaching direction.

The primary goal of the decoder in the present study is to perform binary classification between two task conditions using EEG signals. The classifiers used in previous studies had various designs that differed in feature selection methods and classifier types. In this study we choose to extract spatiotemporal patterns from a set of 30 electrodes that cover the bilateral frontal, central, and parietal areas (Figure 3A). Specifically, we define a standard montage with the electrode order in the data as follows: F1, F3, F5, FC5, FC3, FC1, C1, C3, C5, CP5, CP3, CP1, P1, P3, P5, F2, F4, F6, FC6, FC4, FC2, C2, C4, C6, CP6, CP4, CP2, P2, P4, P6. The spatiotemporal patterns were then classified by linear discriminate analysis (LDA) classifiers. Specifically, the classifications were performed within shifting time windows that were 400 ms long with a shifting step size of 100 ms. This represents a 270-dimensional data vector in a given time window for one trial. We used principal component analysis (PCA) to reduce the dimensionality of the feature space, and we kept the principal components (PC) that explain the first 85% of the variance within the trial pool (see below). The loadings of these PCs formed the feature vectors (approximately 20 dimensional on average) used for training and testing the classifiers. Importantly, we used the following three different decoding configurations.

#### 2.5.1. Within-hand decoding

This is the common configuration used in past research, where trials from a pair of experimental conditions were pooled. In this study, we have eight different experimental conditions as a result of four task conditions performed by either hand. We define them as three-letter codes following the order: Reaching direction, Action type, and Hand. For example, a Left Grasp task performed by the right hand is abbreviated LGR, and a Right Touch task performed by the right hand is RTR. Each of these conditions included 40 trials except those that were excluded. To perform within-hand decoding in each time window, a pool of trials was created from a pair of experimental conditions that differ either in Action type or Reaching direction. The classifier performance was evaluated as the average accuracy from 10 repetitions of 10-fold cross-validation. This configuration allows us to examine the extent to which hand action and reaching direction can be decoded within the same hand (Figure 3B).

#### 2.5.2. Normal cross-hand decoding

To investigate how neural information associated with reach-to-grasp is shared across two hands, we used trials from one hand to train the classifiers which were tested on data from the contralateral hand. Note that determining which conditions can be considered as the same class across two hands is not trivial. Specifically, the class labeling was performed in two ways: *Extrinsic* and *Intrinsic* (Figure 3C). Extrinsic labeling considers spatial congruency in the extrinsic object-centered coordinate. For example, the left hand grasping the right handle (RGL) and the right hand grasping the right handle (RGL) were labeled as the same class for a classifier. In contrast, intrinsic labeling considers joint space congruency in the intrinsic limb-centered coordinate. For example, the left hand touching the right handle (RTL) and the right hand touching the left handle (LTR) were labeled as the same class for a classifier, since the hands were reaching the contralateral side handles. This configuration requires pooling trials from four experimental conditions, two from each hand. The classifier performance was evaluated by using conditions from one hand as the training set and conditions from the other hand as the testing set.

#### 2.5.3. Mirror cross-hand decoding

Given our understanding that motor control for each limb is partially lateralized to the contralateral hemisphere, we also investigated the extent to which spatial patterns of cortical activities may be shared across two hands in a mirrored fashion. We extended the classification configuration of the normal cross-hand decoding described above, by using a mirrored electrode montage for the testing data from the contralateral hand. The mirroring was performed with respect to the midline, which creates a new order: F2, F4, F6, FC6, FC4, FC2, C2, C4, C6, CP6, CP4, CP2, P2, P4, P6, F1, F3, F5, FC5, FC3, FC1, C1, C3, C5, CP5, CP3, CP1, P1, P3, P5. The pooling of trials for each time window remains the same as the normal cross-hand decoding, i.e., from four experimental conditions with either extrinsic or intrinsic class labels. Note that the feature extraction PCA was performed on the trial pool after mirroring the montage. The mirrored montage effectively creates a mirrored spatial distribution of EEG signals, treating left-hemisphere activities as right-hemisphere activities and vice versa for the testing data. In other words, the mirrored montage favors EEG spatial patterns that encode information in a manner that is consistent between two hands across ipsilateral-contralateral hemispheres. In contrast, the standard montage favors EEG spatial patterns that are consistent between two hands across right-left hemispheres.

### 2.6. Statistical analysis

We applied the three decoding configurations to both the planning and execution datasets. The peak decoding accuracy for a given decoding setup (Figure 3; e.g., within-hand action decoding using RGL/RTL conditions) was identified for the same pool of trials across multiple time windows within each dataset. For planning, the start of the time windows spans from the Task cue to 1.5 s post Task cue. For execution, the start of the time windows spans from the movement onsets to 1.5 s post-onset. This led to a total of 15 time windows for each decoding setup in each phase, and the greatest decoding accuracy (and its timing) among these was selected to represent the corresponding decoding setup. We considered a threshold of 59.3% for a peak decoding accuracy to be significantly above the chance level (*p* < 0.05), given that each classifier was tested with approximately 80 total samples (Combrisson and Jerbi, 2015). Statistical comparisons were performed on peak decoding accuracies and the timings of peak decoding accuracies. We first used two-way repeated ANOVA to examine if the peak performance of an Action classifier or a Direction classifier may be different between subsets of data used for training and testing. Since we did not find significant effects of the two factors involved in these ANOVAs, we averaged the results across subsets of data from the same classifier group, and used two-tail paired *t*-tests to compare classifier performance between specific classifier groups.

## 3. Results

### 3.1. Characteristics of limb movements

**FIGURE 4:**
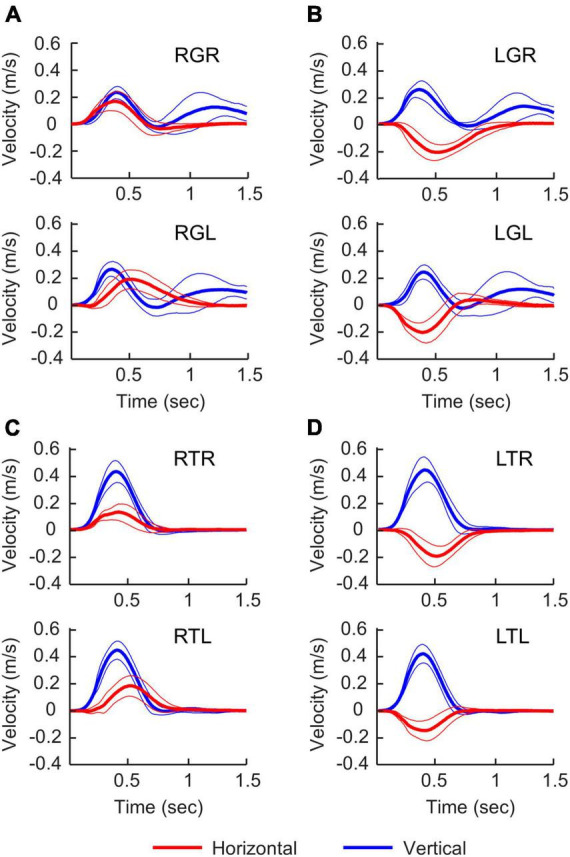
Velocity profiles in different experimental conditions. Right-hand and left-hand profiles are shown at the top and bottom of each panel. Time 0 s is movement onset. Thick and thin lines represent the mean and standard deviation. For horizontal velocities (red), positive values represent moving to the right. For vertical velocities (blue), positive values represent moving upward. **(A)** Grasping the right handle, **(B)** grasping the left handle, **(C)** touching the right handle, **(D)** touching the left handle.

We estimated the limb movement by tracking marker sets attached to the wrist. The movement onsets with respect to the “Go” cue was similar across all experimental conditions (338.5 ± 113.7 ms). There were substantial differences in movement characteristics between the Grasp and Touch conditions starting from the beginning of the movements (Figure 4). This is because the wrist must supinate before contacting the object to align the hand grasp axis with the vertical handle in the Grasp conditions, but not Touch conditions. Furthermore, Grasp conditions feature double peak vertical velocity profiles due to the need to lift the object off the table after reaching. The peak vertical velocity of reaching movement occurred at approximately 0.4 s after movement onset in all experimental conditions, and the object contact occurred at approximately 0.8 s after movement onset. The second vertical velocity peak occurred approximately at 1.2 s after movement onset. Overall, the movement recordings suggest that the motor behavior of the left and right hands were very similar in task conditions that share the same task goals.

### 3.2. Within-hand decoding performance

**FIGURE 5:**
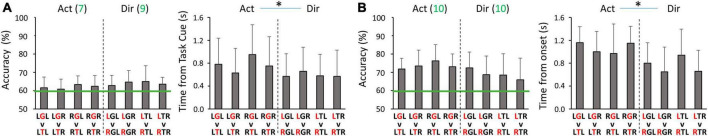
Peak accuracies and the corresponding timing of within-hand decoding **(A)** during the planning phase, and **(B)** during the execution phase. For all panels, Act and Dir denote the Action classifier and Direction classifier groups, respectively. Red letters in the condition code represent the factor being classified. Green lines denote the threshold of 59.3% for above-chance level accuracy. The green values after the classifier type (Act or Dir) in the decoding accuracy panels are the numbers of participants (out of 10) who had above-chance level peak accuracy on average for the corresponding group of four classifiers. Asterisks represent significant differences (*p* < 0.05).

For every group of four binary classifiers that decode the same information (Figure 3B), we did not find any significant differences in peak decoding accuracies or the timings of the peak accuracies. Specifically, classification performance was found to be similar between left and right hands. Therefore, we report the results that are averaged across classifiers from the same group, i.e., Action and Direction decoders. In the planning phase, the peak decoding accuracy was 62.0 ± 3.5% for Action and 64.0 ± 3.9% for Direction, with most participants having above chance level accuracies (Figure 5A). The timing of the peak accuracy was 0.78 ± 0.19 s and 0.60 ± 0.16 s post Task cue for Action and Direction classifiers, respectively. Paired *t*-tests revealed that the peak Direction decoding accuracy occurred significantly earlier than the peak Action accuracy (*p* = 0.045). In the execution phase, the peak decoding accuracy was 73.6 ± 5.7% for Action and 68.9 ± 8.8% for Direction, and all participants had above-chance level peak accuracies (Figure 5B). The timing of the peak accuracy was 1.07 ± 0.22 s and 0.76 ± 0.22 s for Action and Direction classifiers, respectively. A paired *t*-test revealed that the peak Direction decoding accuracy occurred significantly earlier than the peak Action accuracy (*p* = 0.002). Overall, these results suggest that motor intent can be decoded for hand-object interactions and reaching directions with our binary classification setup for both hands.

### 3.3. Normal cross-hand decoding performance

**FIGURE 6:**
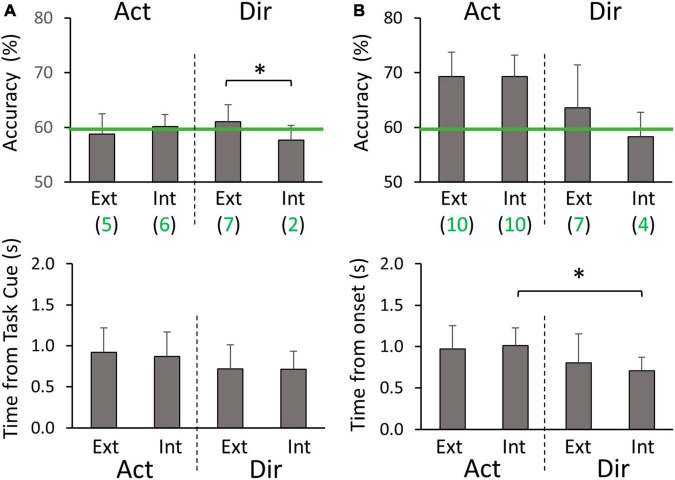
Cross-hand classification peak accuracies and timings **(A)** during the planning phase, and **(B)** during the execution phase. Ext and Int denote extrinsic and intrinsic class labeling methods, respectively. Green lines denote the threshold of 59.3% for above-chance level accuracy. Green values below the labeling method type (Ext or Int) are the numbers of participants (out of 10) who had above-chance level peak accuracy on average for the corresponding group of four classifiers. Asterisks represent significant differences (*p* < 0.05).

In the normal cross-hand decoding configuration, we used the same standard electrode montage for both limbs. Here we focus on comparing the average peak classification performance between different groups of four classifiers (Figure 3C). In the planning phase, we did not find Action decoding to differ between extrinsic and intrinsic labeling (59.5 ± 2.3% on average). Both labeling methods had about half of the participants showing above-chance level accuracy. In contrast, the peak accuracy of Direction decoding was significantly higher with extrinsic labeling than intrinsic labeling (*p* = 0.042), with more participants showing above-chance level accuracy (Figure 6A). No timing differences were found for peak accuracies (0.80 ± 0.13 s post Task cue on average). In the execution phase, Action decoding peak accuracies were similar between extrinsic and intrinsic labeling (69.3 ± 3.5% on average), and all participants were above chance level (Figure 6B). No significance was found between the two labeling methods for Direction decoding peak accuracy, but more participants were above chance level with extrinsic labeling. Additionally, we found that the timing of peak accuracy was earlier for Direction than Action decoding only with intrinsic labeling (*p* = 0.006). In sum, the cross-hand decoding configuration showed that Action classification was not dependent on the class labeling methods, but Direction classification performance favors extrinsic labeling in both planning and execution phases.

### 3.4. Mirror cross-hand decoding performance

**FIGURE 7:**
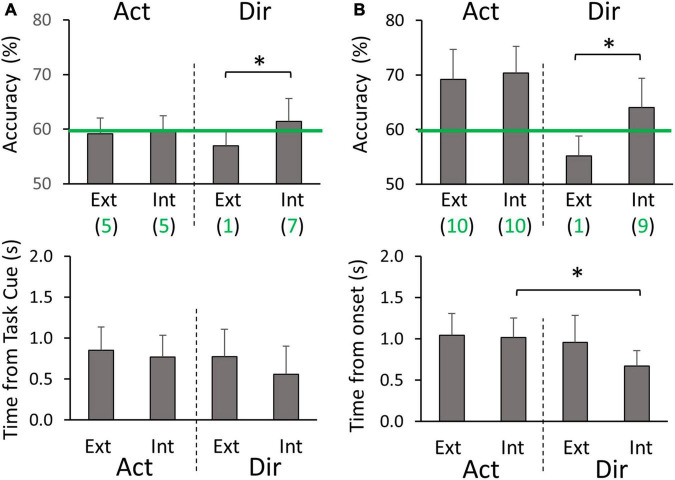
Cross-hand mirror classification peak accuracies and timings **(A)** during the planning phase, and **(B)** during the execution phase. Ext and Int denote extrinsic and intrinsic class labeling methods, respectively. Green lines denote the threshold of 59.3% for above-chance level accuracy. Green values below the labeling method type (Ext or Int) are the numbers of participants (out of 10) who had above-chance level peak accuracy on average for the corresponding group of four classifiers. Asterisks represent significant differences (*p* < 0.05).

In the mirror cross-hand decoding configuration, we used the standard electrode montage for the training limb and mirrored electrode montage for the testing limb. We again focus on comparing the average peak classification performance between different sets of four classifiers (Figure 3C). In the planning phase, we did not find Action decoding to differ between extrinsic and intrinsic labeling (59.4 ± 2.1% on average), which was also at a level similar to the normal cross-hand configuration. For the peak accuracy of Direction decoding, we found it to be opposite to the normal cross-hand configuration. The intrinsic labeling was significantly more accurate than extrinsic labeling (*p* = 0.049), with more participants showing above-chance level accuracy (Figure 7A). No timing differences were found for peak accuracies (0.74 ± 0.14 s post Target cue on average). In the execution phase, Action decoding peak accuracies were again similar between extrinsic and intrinsic labeling (69.8 ± 5.1% on average), and all participants were above chance level (Figure 7B). This result was similar to the normal cross-hand configuration. The Direction decoding performance in this phase followed the same pattern as the planning phase. Intrinsic labeling was found to be significantly more accurate than extrinsic labeling (*p* = 0.001), with more participants above the chance level. Furthermore, we found that the timing of peak accuracy was also earlier for Direction than Action decoding with intrinsic labeling (*p* = 0.003). In sum, the cross-hand mirror decoding configuration showed that Action classification was not dependent on the class labeling methods, but Direction classification performance favors intrinsic labeling in both planning and execution phases.

## 4. Discussion

In this study, we examined the decoding of motor intent for reaching and object interaction using low-frequency component of EEG signals from bilateral frontal-central-parietal areas. It was found that both the intended hand-object interaction and the reaching direction can be decoded with 2-class classifiers during motor planning phase and execution phase, and the peak decoding accuracies for the left and right hands were similar. Furthermore, for the first time, we demonstrated that a classifier trained with EEG data from one limb can decode movement direction and action type for the contralateral limb. Importantly, the success of such cross-hand decoding, especially for movement direction, was dependent on how EEG channels were organized and how movement direction was represented.

### 4.1. Comparing within-hand decoding accuracy to existing research

The experimental setup and classifier designs used in past EEG-based studies vary substantially, which makes direct comparison challenging. The feature vector used in classification can be extracted in different ways to capture spatial, temporal, or spectral patterns. The classifiers can be linear such as LDA and shrinkage LDA, or non-linear such as support vector machines (SVM). Nevertheless, we listed some results from similar studies for decoding hand-object interaction and reaching directions in Tables 1, 2, respectively. Note that the primary goal of the present study was not to compete for higher within-hand decoding accuracy. Instead, by comparing our within-hand configuration to existing research, we can validate our experimental protocol and classifier design to support the main objective of cross-hand decoding.

For hand-object interactions, most studies focused on classifying different grasp types, but they differ in the actions after grasp configurations were formed: some required lifting the object whereas others did not. Generally, the decoding accuracies were higher during motor execution than motor planning or imagery. This is likely due to the need to send efferent information for muscle contraction and the receiving of sensory information during motor execution. Overall, our results are consistent with these studies, showing 2-class decoding accuracy above 60% and above 70% during planning and execution phases for both hands. The relatively high accuracy we achieved in execution may be related to the significant difference between the two hand-object interactions used in our experiment. The grasping condition requires finger force to maintain the grasp, wrist torque to balance the object, and arm force to overcome the object’s weight. In contrast, the upper limb muscle activations were much lower in the touch condition where only a light contact was made between the index fingers and the object.

For reaching directions, most of the existing studies used center-out reaching movements, without a functional goal after reaching is completed. There were more variations in classifier design with some implementing more sophisticated feature selection and classification algorithms. Our decoding accuracies during both planning and execution for the left and right hands were lower than those from previous studies. One explanation is that our classifier is relatively simple without capturing spectral information or creating non-linear decision boundaries, which may lower the ability to discriminate information related to reaching target representations. Another potential cause is that some of the previous studies did not use EOG to remove artifacts associated with eye movements, which can be highly predictive for target visual locations. Nevertheless, our decoding accuracies for movement direction were still significantly above the chance level. One important feature of our experimental task is that participants must perform distinct hand actions after reaching the object in the Grasp conditions, i.e., balancing a left-heavy or right-heavy object depending on which handle was grasped. However, such follow-up actions did not significantly increase the peak decoding accuracy in comparison to Touch conditions where different hand actions were not needed. Based on the timing of the peak accuracy, the peak decoding accuracy for task direction during execution was approximately aligned with the end point of reach for both the Grasp and Touch conditions, but before the final stage of sustained object interactions (Figure 4). Furthermore, the timings of the peak decoding accuracy for action were later than the timings for direction decoding in both planning and execution phases. These results indicate that the directional information was mostly associated with the reach movement, independent of the subsequent hand-object interactions.

### 4.2. Decoding hand actions with classifiers trained using data from the contralateral hand

Our results show that cross-hand decoders for discriminating between Grasp and Touch conditions were above chance level in some participants during the planning phase, and in all participants during the execution phase. Interestingly, we did not find any significant difference between different class labeling methods (i.e., extrinsic or intrinsic), or whether mirror electrode montage was used for the contralateral hand. The lack of dependence on class labeling indicates that the discriminative information in the classifier was unlikely to be associated with the actual torque direction the hand was exerting during the Grasp conditions. Otherwise, the opposite hand torque direction required by the two handles may favor either extrinsic or intrinsic labeling to discriminate against the Touch condition. For example, for the left-hand training pair LGL/LTL, if the EEG signals carry intrinsic information of the torque direction, i.e., supination, the intrinsic labeling of testing pair RGR/RTR would yield higher accuracy because the extrinsic testing pair LGR/LTR had incongruent hand torque representation (pronation). Moreover, the fact that mirroring electrode montages did not affect decoding accuracy suggests that the spatial distribution of the discrimination-supporting EEG patterns may be symmetric about the midline, which indicates a lack of hemispheric lateralization.

One possible explanation of our results is that the classifier may capture the neural signatures of a bilateral network that encodes hand actions. Indeed, functional magnetic resonance imaging (fMRI) studies revealed that many nodes within the bilateral frontoparietal areas in humans, such as posterior parietal cortex and dorsal premotor cortex, allow limb-independent discrimination between grasping and touch (Gallivan et al., 2013) or between power and precision grasps (Turella et al., 2020). However, the same studies also showed that there are areas where hand action related neural activities are more limb-dependent. An important note is that the BOLD signals generated using fMRI do not directly translate to EEG patterns. The lateralized cortical activities in fMRI may not be strong enough to provide discrimination power as EEG signal sources for hand action classification in the low-frequency band we used. An alternative explanation of the present result is that the peak action decoding may partially depend on neural processes that are not specifically related to the type of hand posture. Note that the peak accuracy occurred during the sustained action phase where grasping required keeping the balance of the object and touching required light touch. It is possible that the grasping conditions is associated with brain regions that are critical to online error monitoring and error correction, such as anterior cingulate (Carter et al., 1998; Kerns et al., 2004) and supplementary motor area (Coull et al., 2016). Both of these areas are aligned with the midline and could lead to symmetrical distributions of EEG signals.

### 4.3. Decoding reaching directions with classifiers trained using data from the contralateral hand

Unlike hand action decoding, the cross-hand classification performance of reaching direction showed clear dependency on both the class labeling and electrode montage. Specifically, extrinsic labeling was superior in normal cross-hand configuration, whereas intrinsic labeling was superior in cross-hand mirror configuration. These findings indicate that the extrinsic representation of reaching direction is likely to be encoded by EEG patterns that are lateralized in a spatially consistent fashion that is independent of the limb performing the task, i.e., biased toward the right- or left-hemisphere. In contrast, the intrinsic representation of the reaching direction is likely to be encoded by EEG patterns that are consistently organized in the ipsilateral-contralateral direction with respect to the limb performing the task. A recent EEG study found that decoding reaching direction for the right limb performing reaching in two different arm postures was more accurate using extrinsic target labeling than intrinsic target labeling (Yoshimura et al., 2017). Our result is consistent with this study, although it did not examine cross-hand decoding. Non-human primate research has shown that neurons across the frontoparietal network are tuned to reaching targets in both extrinsic and intrinsic coordinates. A representation gradient may exist such that parietal regions and premotor regions are more likely to encode reaching direction extrinsically for both arms, whereas primary motor areas are more likely to encode reaching direction intrinsically for the contralateral arms (Kakei et al., 1999, 2001; Cisek et al., 2003; Chang et al., 2008). Human fMRI studies also support such gradient (Bernier and Grafton, 2010; Cavina-Pratesi et al., 2010). Based on these studies, we speculate that the decoding of the extrinsic reaching direction in the present study may rely on information carried in the parietal and premotor regions, particularly from the dominant (right) hemisphere. In fact, it has been theorized that the dominant hemisphere may be more involved in motor attention and predictive control of limb dynamics (Rushworth et al., 2003; Serrien et al., 2006; Sainburg, 2014), which are prominent features of our experimental task. In contrast, we speculate that the decoding of the intrinsic reaching direction in our study may mainly rely on signals originating from the limb-dependent representations in primary motor areas contralateral to the limb being used.

### 4.4. Limitations and future work

We recognize several limitations in the present study. First, we only examined the low-frequency band of the EEG signals using a simple linear feature extraction method and classifiers. Although the method we chose was very common in previous research, there could be alternative classifier designs that can yield higher classification accuracy. Second, we were not able to perform reliable source analysis because we only had a small number of participants, and we did not have the tools to acquire electrode location data and MRI scans which are critical to perform source localization. This prevented us from identifying location information of the neural mechanisms underlying our results. Third, we only used two hand action types and two reaching directions. In real-world BMI applications where a broad range of motor actions needs to be implemented, this may not be sufficient to effectively control assistive or rehabilitation devices. We will address these limitations in future experiments by improving our methodology. Furthermore, EEG decoding of movement intention was not limited to the classification of movement types. It has been shown that arm and finger kinematics can also be predicted by EEG signals (Agashe et al., 2015; Robinson and Vinod, 2016). Therefore, we also plan to investigate the extent to which cross-hand decoding of movement kinematics is viable.

## 5. Conclusion

In the present study, we demonstrated that EEG signals could carry some information about upper limb movement that is shared between two limbs, which enables classifiers trained from one limb to decode the motor intent of the contralateral limb. Furthermore, our findings provide new insights into the visuomotor transformation process underlying reaching and hand-object interactions by comparing extrinsic and intrinsic labeling methods and electrode montages. Future work is needed to examine how these findings can be used to improve BMIs by allowing fast calibration processes or more robust decoding algorithms.

## Data availability statement

The raw data supporting the conclusions of this article will be made available by the authors, without undue reservation.

## Ethics statement

The studies involving humans were approved by the Institutional Review Board at University of Central Florida. The studies were conducted in accordance with the local legislation and institutional requirements. The Ethics Committee/Institutional Review Board waived the requirement of written informed consent for participation from the participants or the participants’ legal guardians/next of kin because the risk was considered to be minimal.

## Author contributions

KH: Conceptualization, Data curation, Formal analysis, Investigation, Methodology, Validation, Visualization, Writing – original draft, Writing – review and editing. RE-S: Investigation, Methodology, Validation, Writing – review and editing. QF: Conceptualization, Formal analysis, Funding acquisition, Methodology, Project administration, Software, Supervision, Validation, Visualization, Writing – original draft, Writing – review and editing.
